# Deep Learning to Identify Cognitive Impairment Across the Full Spectrum of Chronic Kidney Disease: Stage- and Cognition-Informed Feature Selection with a Dual-Branch Feature-Gated Network

**DOI:** 10.34133/cbsystems.0733

**Published:** 2026-11-06

**Authors:** Wenbo Yang, Lijun Song, Hang Lei, Shiqing Wang, Xintian Ren, Yanfang Tan, Boyan Xu, Zhenghan Yang, Min Li, Weikang Guo, Anfu Zhou, Zhenchang Wang, Hao Wang

**Affiliations:** ^1^ Department of Radiology, Beijing Friendship Hospital, Capital Medical University, Beijing 100050, China.; ^2^ State Key Laboratory of Networking and Switching Technology, Beijing University of Posts and Telecommunications, Beijing 100876, China.; ^3^ Clinical Epidemiology and EBM Unit, Beijing Friendship Hospital, Capital Medical University, Beijing 100050, China.; ^4^ MR Research, GE Healthcare, Beijing 100176, China.; ^5^ Department of Nephrology, Beijing Friendship Hospital, Capital Medical University, Beijing 100050, China.; ^6^ Precision and Intelligence Medical Imaging Lab, Beijing Friendship Hospital, Capital Medical University, Beijing 100050, China.

## Abstract

**Introduction:** Cognitive impairment (CI) is a frequent and clinically relevant complication of chronic kidney disease (CKD), yet the neuroimaging features associated with CI remain incompletely characterized. This study developed a stage- and cognition-informed neuroimaging feature-selection framework and integrated the selected features into a Dual-Branch Feature-Gated Network (DBFG-Net) for identifying CKD with CI (CKD-CI). **Methods:** We enrolled a discovery cohort of 342 patients with CKD, including 177 with CI and 165 without CI, and 58 age- and sex-matched healthy controls. Multimodal magnetic resonance imaging features were extracted from T1-weighted imaging, arterial spin labeling, and quantitative susceptibility mapping. Stage-related imaging patterns were first characterized across CKD stages 1 to 5, followed by cognition-related comparisons between CKD-CI and CKD without CI. Features showing both stage-related linear trends and cognition-related discrimination were selected for DBFG-Net training and tested in temporal and multicenter external validation cohorts. **Results:** Structural morphology and tissue susceptibility showed significant stage-related linear trends across CKD stages, whereas cerebral blood flow showed no significant stage-related linear trend. Consistently, discriminative features for CKD-CI were confined to structural and susceptibility measures. DBFG-Net achieved area-under-the-receiver-operating-characteristic-curve values of 0.942 in the discovery cohort and 0.902 and 0.926 in the 2 external validation cohorts. **Conclusion:** The stage- and cognition-informed framework identified structural and susceptibility features relevant to CKD-CI. DBFG-Net demonstrated robust performance for CKD-CI identification across the discovery and external validation cohorts.

## Introduction

Chronic kidney disease (CKD) represents a major global public health burden, with a prevalence of approximately 14.2% [1,2]. A considerable proportion of patients experience progressive estimated glomerular filtration rate (eGFR) decline, which may ultimately lead to end-stage renal disease (ESRD) [3,4]. Cognitive impairment (CI), a major complication of CKD, spans a continuum from subtle deficits in executive function, attention, and memory to overt dementia [5]. This impairment profoundly diminishes health-related quality of life, compromises treatment adherence, and worsens clinical outcomes [6]. However, the mechanisms underlying CI in CKD remain incompletely understood. Several pathways linking kidney dysfunction to brain injury have been proposed to explain this cognitive decline, including uremic toxin accumulation, vascular injury, chronic inflammation, metabolic dysregulation, and accelerated brain aging [7,8].

Multimodal magnetic resonance imaging (MRI) provides a noninvasive approach to characterize brain alterations in patients with CKD [9,10]. Different MRI modalities capture complementary aspects of brain integrity, including structural morphology, cerebral perfusion, and tissue susceptibility [11]. High-resolution T1-weighted imaging enables the quantitative assessment of morphometric indices such as cortical thickness and gray-matter volume. Based on these metrics, previous studies have reported widespread cerebral atrophy in patients with CKD, particularly those with ESRD [12,13]. Arterial spin labeling (ASL) allows the noninvasive measurement of cerebral blood flow (CBF). Prior studies have shown abnormal perfusion patterns in patients with advanced CKD or ESRD [14–16]. Quantitative susceptibility mapping (QSM) provides susceptibility measures related to iron deposition and myelin content [17]. Increased susceptibility in deep gray matter has been associated with poorer cognitive performance in patients with ESRD [18,19]. Together, these findings suggest that multimodal neuroimaging features may serve as potential imaging markers of brain injury and CI in CKD. Nevertheless, existing findings remain heterogeneous, likely due to differences in CKD stage distribution, imaging modalities, and analytic strategies.

Previous neuroimaging studies in CKD have primarily focused on ESRD, leaving brain alterations across the full spectrum of CKD insufficiently characterized. As CKD advances, brain abnormalities may evolve with disease progression. Therefore, stage-related neuroimaging changes may provide important context for interpreting CI-related alterations. Deep learning has been increasingly applied to biomedical imaging for automated analysis, multimodal information integration, and disease classification [20–22]. Integrating CKD stage-related neuroimaging patterns with cognition-related differences may help identify diagnostically informative features for deep-learning models. By reducing noisy or redundant inputs, this strategy may mitigate overfitting, improve interpretability, and facilitate more reliable CI identification in patients with CKD.

In this study, we aimed to develop a deep-learning model for the identification of CI in patients with CKD using a stage- and cognition-informed neuroimaging feature-selection strategy. Specifically, this study was designed to (a) characterize CKD stage-related neuroimaging patterns using multimodal MRI; (b) identify cognition-related alterations overlapping with stage-related trend features; and (c) integrate the selected features into the Dual-Branch Feature-Gated Network (DBFG-Net) for robust CI identification.

## Materials and Methods

### Participants

This study was approved by the Medical Ethics Committee of Beijing Friendship Hospital, Capital Medical University (approval number: 2022-P2-359-01), which served as the primary approval shared across all participating centers. All participants provided written informed consent prior to enrollment.

The study design, cohorts, and analytic workflow are summarized in Fig. 1. This study comprised a discovery cohort and 2 external validation cohorts. Patients with CKD were prospectively enrolled according to the National Kidney Foundation–Kidney Disease Outcomes Quality Initiative guidelines, with inclusion criteria including age 18 to 70 years and right-handedness. Exclusion criteria encompassed (a) history of cerebrovascular or intracranial disease (e.g., stroke, traumatic brain injury, or intracranial tumor); (b) renal replacement therapy; (c) substance or alcohol abuse; (d) any psychiatric disorder; (e) MRI contraindications; and (f) poor MRI image quality or incomplete imaging data.

**Fig. 1.**
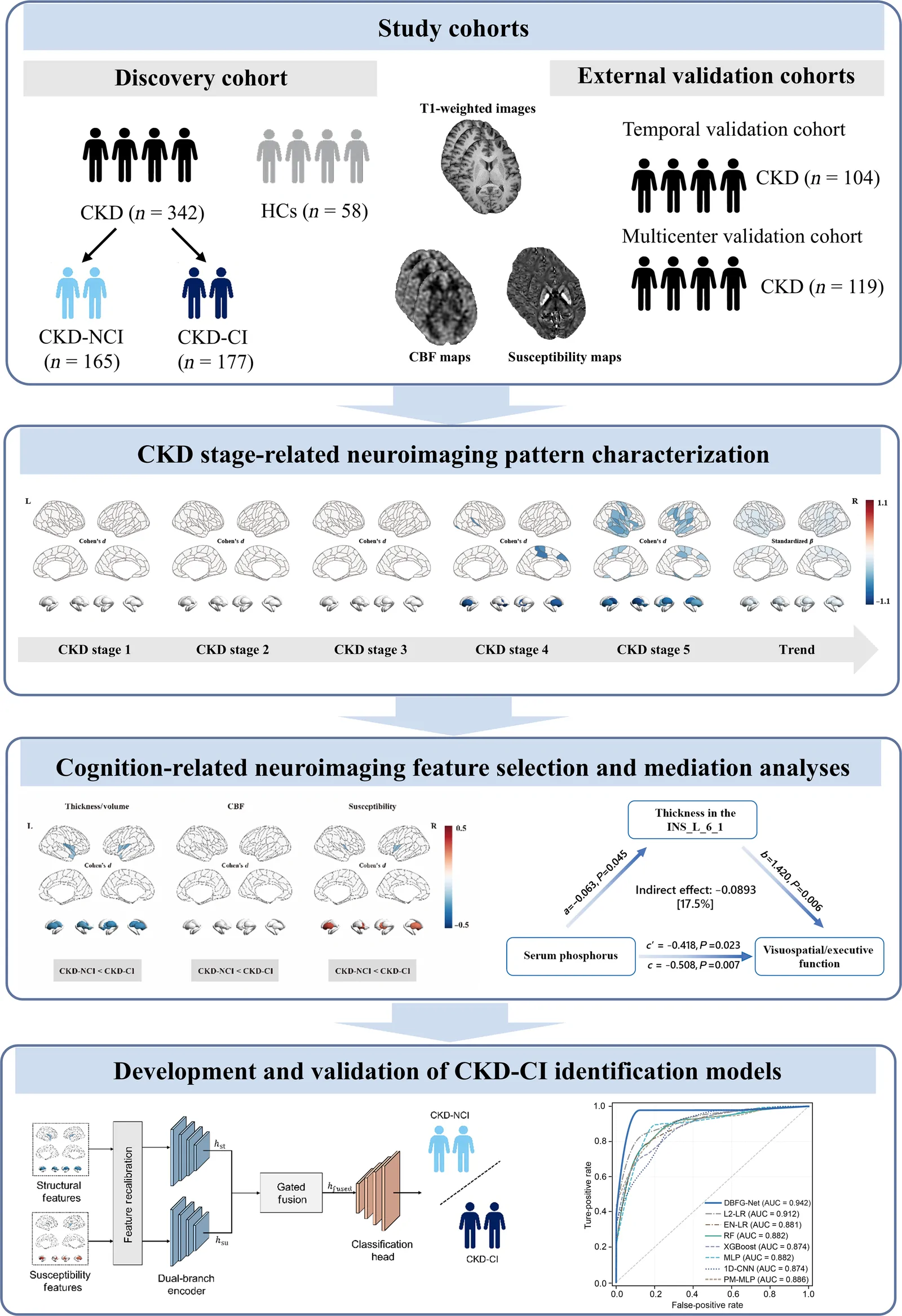
Study design, cohorts, and analytic workflow. CKD, chronic kidney disease; HCs, healthy controls; CKD-CI, chronic kidney disease with cognitive impairment; CKD-NCI, chronic kidney disease without cognitive impairment; CBF, cerebral blood flow; INS_L_6_1, left insular part 1; DBFG-Net, Dual-Branch Feature-Gated Network; AUC, area under the receiver operating characteristic curve; L2-LR, L2-regularized logistic regression; EN-LR, elastic-net logistic regression; RF, random forest; XGBoost, extreme gradient boosting; MLP, multilayer perceptron; 1D-CNN, one-dimensional convolutional neural network; PM-MLP, parameter-count-matched multilayer perceptron.

The discovery cohort consisted of 342 CKD patients (CKD with CI [CKD-CI], *n* = 177; CKD without CI [CKD-NCI], *n* = 165) classified according to education-adjusted Montreal Cognitive Assessment (MoCA) scores (CKD-CI: adjusted MoCA < 26; CKD-NCI: adjusted MoCA ≥ 26). The discovery cohort also included 58 age- and sex-matched healthy controls (HCs) recruited via community advertisement and screened using identical exclusion criteria. The discovery dataset was used to characterize multimodal MRI brain alterations in CKD. For model development and internal validation, only CKD patients from the discovery cohort were used (CKD-CI vs. CKD-NCI), excluding HCs from training.

To assess model generalizability, 2 external validation cohorts were established: (a) a temporal validation cohort of 104 CKD patients (CKD-CI, *n* = 42; CKD-NCI, *n* = 62) from the same center as the discovery cohort and (b) a multicenter validation cohort of 119 CKD patients (CKD-CI, *n* = 46; CKD-NCI, *n* = 73) from 2 independent centers. Detailed enrollment timelines, specific exclusion numbers, and center information are provided in Supplementary Methods.

### Clinical, laboratory, and cognitive assessments

Demographic characteristics including age, sex, and years of education were collected for all participants. Clinical and laboratory data were obtained concurrently, including serum creatinine, urea, hemoglobin, total cholesterol, triglycerides, serum iron, serum calcium, and serum phosphorus. In addition, eGFR was calculated using the 2021 Chronic Kidney Disease Epidemiology Collaboration creatinine equation [23]. Cognitive function was assessed using the MoCA (Beijing version). To account for educational attainment, 1 point was added to the raw MoCA total score for participants with ≤12 years of formal education, with the adjusted total score capped at 30.

### MRI data acquisition

All MRI examinations in the discovery and external validation cohorts were performed on 3.0-T scanners (Discovery MR750w; GE Healthcare, Milwaukee, WI, USA) equipped with an 8-channel phased-array head–neck coil. Participants were instructed to remain still with eyes closed and ears plugged during all acquisitions. Multimodal data included structural T1-weighted images, ASL, and QSM data. Detailed acquisition sequences and parameters are provided in Supplementary Methods.

### MRI data processing

Structural T1-weighted images were processed using FreeSurfer (v7.4.1) with the standard recon-all pipeline, including motion correction, Talairach transformation, skull stripping, intensity normalization, tissue segmentation, and surface reconstruction [24].

CBF maps were generated using GE FuncTool software based on a single-compartment model for pseudo-continuous ASL [25]. CBF images were subsequently co-registered to individual T1-weighted images in native space using boundary-based registration with white-matter refinement as implemented in FSL (v6.0.7).

QSM data were processed using the SEPIA software package to generate susceptibility maps, following the recommended steps outlined in the consensus paper [26]. Briefly, phase images were unwrapped using a Laplacian-based approach, background fields were removed using V-SHARP, and susceptibility inversion was performed using MEDI. The resulting susceptibility maps were first linearly registered to native T1-weighted space and then refined with nonlinear registration using ANTs (v2.5.3).

All multimodal metrics were extracted in native T1-weighted space according to the Brainnetome atlas, which parcellates the brain into 246 regions, including 210 cortical and 36 subcortical subregions [27]. For subcortical analyses, the 36 original subregions were merged into 14 regions to improve statistical robustness (Table S1). In total, 224 regions of interest (ROIs) were included in the analyses (210 cortical and 14 subcortical). Mean cortical thickness, CBF, and susceptibility values were calculated for each of the 210 cortical regions. Regional volumes, mean CBF, and mean susceptibility were computed for the 14 merged subcortical regions.

### Stage- and cognition-informed feature selection and normalization

In the discovery cohort, we implemented a stage- and cognition-informed feature-selection strategy to identify neuroimaging features for subsequent model construction. First, within each imaging modality, stage-related neuroimaging patterns were assessed across CKD stages using all ROI-level features. Features showing significant linear trends across CKD stages were defined as stage-sensitive neuroimaging features. Second, independently of the stage-related trend analysis, cognition-related group differences were evaluated between CKD-CI and CKD-NCI patients across all neuroimaging features. Only features that simultaneously showed significant stage-related linear trends and significant cognition-related group differences were retained as candidate features for model construction.

For internal validation, the complete feature-selection procedure was repeated independently within each outer development set using only the data available in that set. The held-out outer test fold was not involved in either the stage-related trend analysis or the CKD-CI versus CKD-NCI comparison. Therefore, the number and identity of selected features were allowed to vary across outer folds. After completion of internal validation, the same feature-selection procedure was reapplied to the complete discovery cohort to define the final feature set for external validation. This procedure identified 24 neuroimaging features, including 17 structural and 7 susceptibility features. These features were subsequently fixed for final model development and independent external validation. No feature selection or feature re-selection was performed in the external validation cohorts.

Because neuroimaging features derived from different brain regions and modalities varied substantially in numerical range and measurement scale, the selected ROI-level features used for model input were standardized using *Z*-score normalization. During internal validation, normalization parameters were estimated exclusively from the corresponding training data and subsequently applied to the validation and held-out test data. For external validation, normalization parameters derived from the complete discovery cohort were applied unchanged to the external cohorts. No normalization parameters were re-estimated using external validation data.

### Model architecture

The proposed DBFG-Net was designed to identify CI based on the selected neuroimaging features. The overall workflow of DBFG-Net is illustrated in Fig. 2. Guided by neuroimaging prior knowledge, DBFG-Net first recalibrated the selected neuroimaging features and then separately encoded structural and susceptibility features using 2 independently parameterized modality-specific branch encoders. The resulting branch-specific embeddings were subsequently integrated through a gated fusion module for CI identification.

**Fig. 2.**
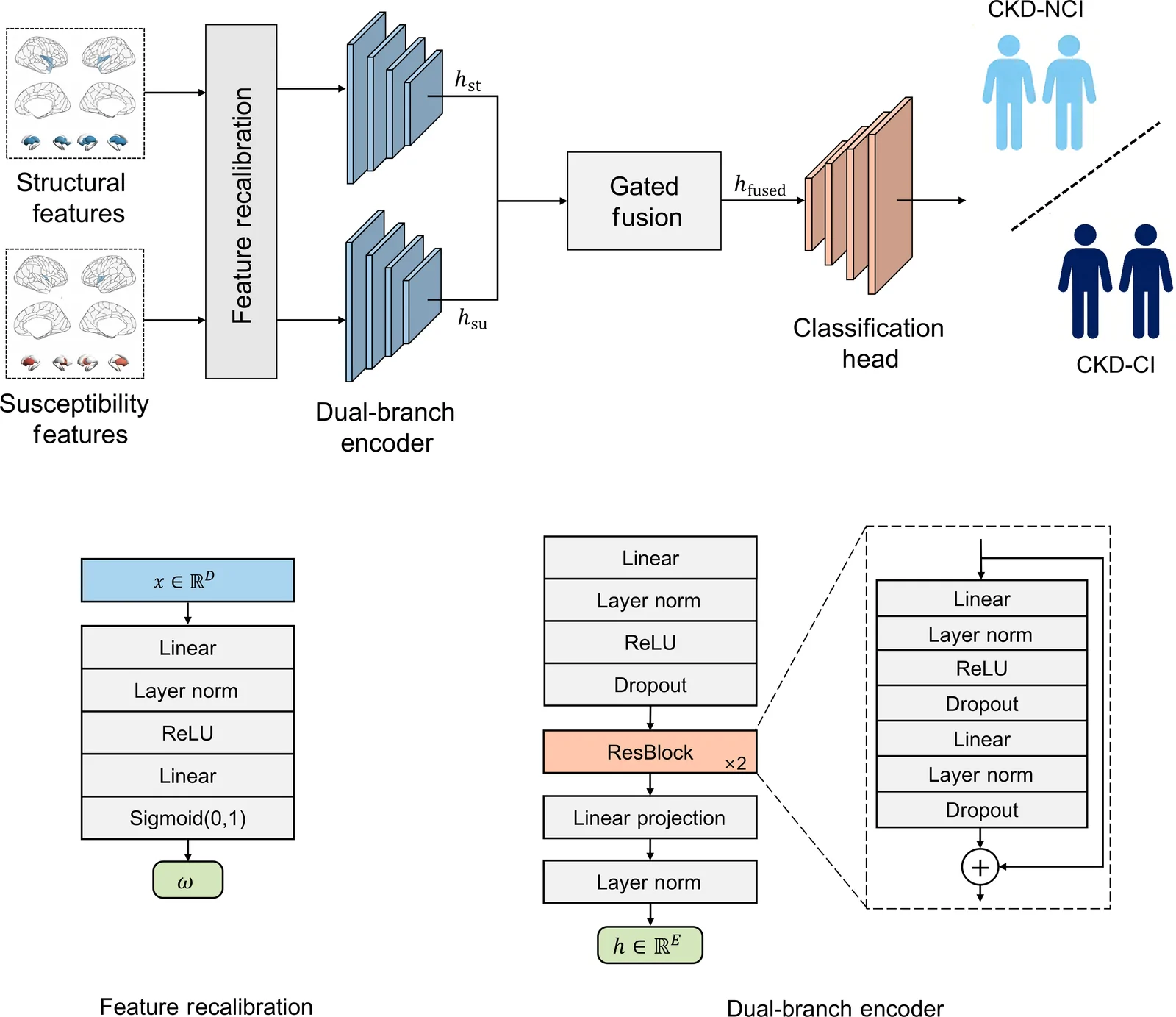
Architecture of the DBFG-Net. ReLU, rectified linear unit.

The input to DBFG-Net consisted of the neuroimaging features selected within the corresponding training data, denoted as $x\in {\mathbb{R}}^{D}$ , where D represents the number of selected features. The selected features were separated into structural and susceptibility branches according to imaging modality. For the final model used for external validation, D=24 , comprising 17 structural and 7 susceptibility features.

Before branch-wise encoding, a lightweight feature-recalibration module was applied to adaptively learn the importance of each input feature. Specifically, the input vector was first projected from the original dimension $\left(D\right)$ to a hidden dimension $\left(H=12\right)$ , followed by layer normalization and rectified linear unit (ReLU) activation. The hidden representation was then mapped back to the original input dimension, and a sigmoid activation function was used to generate a feature-wise weight vector $w\in {\left(0,1\right)}^{D}$ . The recalibrated feature representation was obtained by element-wise multiplication between the learned weight vector and the original input features.

The recalibrated structural and susceptibility features were subsequently fed into 2 modality-specific encoders. The 2 branches shared the same architectural design but used independent parameters to capture modality-specific feature distributions and discriminative patterns. Each branch first performed feature mapping through a feed-forward multilayer perceptron (MLP) backbone with hidden layer dimensions of 64 and 32. Each layer consisted of a linear transformation followed sequentially by layer normalization, ReLU activation, and dropout. Following the feed-forward backbone, each branch further stacked 2 residual MLP blocks. Each residual block contained 2 fully connected layers, each followed by layer normalization, ReLU activation, and dropout. A skip connection added the block input to the transformed output, followed by a ReLU activation to produce the block-level output.

At the output of the branch encoders, an embedding projection layer generated fixed-dimensional embedding representations, denoted as $h_{\mathrm{st}},h_{\mathrm{su}}\in {\mathbb{R}}^{32}$ . The output embeddings were subsequently normalized using layer normalization and passed to the gated fusion module. The gated fusion module was designed to adaptively integrate the embeddings from the 2 branches. The 2 embeddings were first concatenated and then passed through a lightweight MLP to generate a scalar gating weight $g\in \left(0,1\right)$ . The fused representation was computed as

$$h_{\text{fused}}=g\cdot h_{\mathrm{st}}+\left(1-g\right)\cdot h_{\mathrm{su}}$$ (1)

The fused features were subsequently fed into the classification head for prediction. The classification head was implemented as an MLP, with hidden layer dimensions set to 32 and 16. Each hidden layer was followed by a ReLU activation function and a dropout operation. The output layer was a single fully connected neuron that produced a scalar logit for binary classification. During training, the model was optimized using binary cross-entropy loss with logits.

Under the final 24-feature configuration, DBFG-Net contained 19,646 trainable parameters.

### Model training and internal validation

The proposed DBFG-Net was implemented in Python (v3.11) using the PyTorch framework (v2.1.0). Internal model performance was evaluated using 5-fold cross-validation. Participants in the discovery cohort were randomly partitioned into 5 nonoverlapping outer folds, with approximately 20% of participants held out as an independent test set in each iteration. The remaining approximately 80% constituted the outer development set, which was further split, using stratified random sampling, into a training set (80% of the development set) and an internal validation set (20% of the development set). The validation set was used for model and checkpoint selection, whereas the outer test set remained unseen until final evaluation. Model hyperparameters, including the network hidden layer dimensions, dropout ratio, learning rate, and weight decay, were predetermined before the cross-validation procedure.

Network parameters were optimized using the Adam optimizer with a learning rate of 1 × 10^−4^, weight decay of 1 × 10^−4^, and a batch size of 32. Training was performed for a maximum of 200 epochs. The checkpoint with the lowest validation binary cross-entropy loss was retained, with validation accuracy used to resolve ties. Given the relatively balanced class distribution in the training data, the classification probability threshold was fixed at the standard value of 0.50 for binary classification. For each fold, the selected checkpoint was evaluated on the corresponding held-out test fold. Repeating the procedure across all 5 folds generated exactly one out-of-fold (OOF) prediction for every participant. Internal performance was calculated exclusively from the pooled OOF predictions across the 5 held-out folds. All experiments were conducted with fixed random seeds for data splitting and model parameter initialization to ensure reproducibility.

### External validation and comparative models

After completion of internal validation and fixation of the model-development procedure, the same feature-selection strategy was reapplied to the complete discovery cohort, yielding the final set of 24 neuroimaging features. *Z*-score normalization parameters for the final predictive pipeline were estimated from the complete discovery cohort. The final model was trained on the complete discovery cohort for a duration determined from internal cross-validation, without further checkpoint selection. The model was then evaluated in the independent external validation cohorts without further feature selection, model optimization, or outcome-guided adjustment.

For comparative evaluation, 4 conventional machine-learning models, including L2-regularized logistic regression (L2-LR), elastic-net logistic regression (EN-LR), random forest (RF), and extreme gradient boosting (XGBoost), were implemented along with 3 neural-network baselines, namely, an MLP, a one-dimensional convolutional neural network (1D-CNN), and a parameter-count matched MLP (PM-MLP). Comparative models were evaluated using the same fold-specific selected features and data partitions as DBFG-Net. Detailed parameters for all models are provided in Supplementary Methods.

### Additional model analyses

To further evaluate the contribution of the proposed architecture, the robustness of imaging-based prediction, and the incremental value of clinical information, we performed a series of complementary ablation, sensitivity, and stability analyses.

First, architectural ablation analyses were performed by independently removing the feature-recalibration module, gated fusion module, or residual MLP blocks from DBFG-Net. Input-modality ablation analyses were additionally conducted using structural features alone and susceptibility features alone. All ablated models were trained and evaluated using the same analysis framework as the full DBFG-Net.

Second, a disease-severity-adjusted sensitivity analysis was performed to determine whether model discrimination was primarily attributable to overall CKD severity. CKD stage was used as the primary indicator of disease severity. For each selected imaging feature, the component associated with CKD stage was removed using regression-based residualization, and DBFG-Net was subsequently retrained using the residualized imaging features. During internal validation, the residualization model was estimated using training data only and then applied to the corresponding validation and test data.

Third, clinical-only models were constructed using age, sex, years of education, eGFR, serum creatinine, urea, hemoglobin, total cholesterol, triglycerides, serum iron, serum calcium, and serum phosphorus. L2-LR, RF, and XGBoost were used as classifiers. Clinical-imaging models were further constructed using a late-fusion strategy. The imaging risk score generated by DBFG-Net was combined with the same clinical variables and entered into L2-LR, RF, or XGBoost classifiers. Within each outer training set, an additional cross-fitting procedure was used to generate OOF imaging risk scores for training the second-stage classifier. Each training participant’s imaging score was generated by a DBFG-Net model that had not been trained using that participant.

Finally, DBFG-Net stability was assessed in the discovery cohort using 10 prespecified random seeds (7, 19, 42, 73, 101, 202, 404, 808, 2,024, and 2,026). In the first analysis, the original 5-fold allocation was held fixed while only training randomness was varied. In the second analysis, stratified 5-fold partitions were regenerated for each seed, with feature selection and *Z*-score normalization repeated within each newly generated training partition. Each repeat generated one complete OOF prediction set for all discovery-cohort participants.

### Post hoc interpretability analysis

Model predictions in the external validation cohorts were interpreted using SHapley Additive exPlanations (SHAP). Signed SHAP summary plots were used to visualize the magnitude and direction of feature contributions. Global feature importance was calculated as the mean absolute SHAP value for each feature and normalized to a 0-to-1 scale. The top 10 features were ranked based on their average normalized importance.

### Model evaluation

Model performance was evaluated using the area under the receiver operating characteristic curve (AUC), accuracy, sensitivity, specificity, precision, and F1 score. Confusion matrices were constructed to summarize true-positive, true-negative, false-positive, and false-negative classifications and to visualize class-specific prediction performance. The Brier score was additionally calculated to evaluate the accuracy of predicted probabilities. Ninety-five percent confidence intervals for performance metrics were estimated using bootstrap resampling with 5,000 iterations. Differences in AUC between DBFG-Net and other models, where applicable, were assessed using the DeLong test, and statistical significance was set at *P* <0.05. Clinical utility was further evaluated using decision curve analysis (DCA) with net benefit calculated across a range of probability thresholds.

### Statistical analysis

Statistical analyses were performed in R (v4.5.0). Normality was assessed using the Shapiro–Wilk test. Age and education were compared across HCs and CKD stages using analysis of variance (ANOVA), whereas sex distribution was compared using the chi-square test. Laboratory measures and cognitive scores were compared across CKD stages using the Kruskal–Wallis test. Linear trends were evaluated using linear models, with CKD stage coded as an ordinal numeric variable from 1 to 5. For demographic, laboratory, and cognitive analyses, statistical significance was set at *P* <0.05 without correction for multiple comparisons.

Neuroimaging measures were analyzed using analysis of covariance (ANCOVA) models adjusted for age, sex, education, total intracranial volume, and hemoglobin. Stage-wise analyses compared each CKD stage with HCs, with post hoc pairwise comparisons based on estimated marginal means. Linear trends across CKD stages were assessed using linear models, with CKD stage coded as an ordinal numeric variable from 1 to 5. Effect sizes were reported as Cohen’s *d* for pairwise comparisons and standardized regression coefficients (*β*) for trend analyses. Group differences between CKD-CI and CKD-NCI were also evaluated using ANCOVA models adjusted for age, sex, education, total intracranial volume, and hemoglobin. False discovery rate (FDR) correction was applied to neuroimaging analyses.

Spearman partial correlations were used to examine associations between clinical and cognitive measures, adjusting for age, sex, and education. FDR correction was applied to correlation analyses. Mediation analyses were performed to examine whether neuroimaging measures statistically mediated associations between clinical and cognitive measures, adjusting for age, sex, and education. Indirect effects and their 95% confidence intervals were estimated using 5,000 bootstrap resamples. Mediation analyses were exploratory and were not corrected for multiple comparisons.

## Results

### Participant characteristics

Participant demographics were similar across groups, with no significant differences in age, sex distribution, or years of education. Laboratory measures differed across CKD stages, except for serum calcium. Across CKD stages, eGFR, hemoglobin, total cholesterol, triglycerides, and serum iron decreased, whereas serum creatinine, urea, and serum phosphorus increased. Serum calcium showed no significant trend across stages. The total MoCA score showed a significant negative linear trend across CKD stages. Visuospatial/executive, naming, attention, and language subdomain scores also decreased across stages, whereas abstraction, delayed recall, and orientation did not show significant trends (Table 1).

**Table 1.** Demographic, clinical, and cognitive characteristics across HCs and CKD stages. Test statistic denotes the *F* (ANOVA), *χ* ^2^ (chi-square), or *H* (Kruskal–Wallis) statistic, as appropriate. Trend effect indicates the standardized regression coefficient (*β*) from linear models across CKD stages; Trend *P* is the corresponding *P* value.

Characteristic	HCs (*n* = 58)	CKD stage 1 (*n* = 61)	CKD stage 2 (*n* = 90)	CKD stage 3 (*n* = 69)	CKD stage 4 (*n* = 19)	CKD stage 5 (*n* = 103)	Test statistic	*P*	Trend effect	Trend *P*
Age/y	50.8 ± 8.8	49.7 ± 12.0	51.5 ± 14.2	53.4 ± 11.6	51.9 ± 13.2	51.8 ± 12.8	0.7	0.633 ^a^	–	–
Gender (male/female)	35/23	29/32	59/31	45/24	10/9	69/34	8.0	0.154 ^b^	–	–
Education/y	12.0 ± 4.7	12.4 ± 3.3	12.1 ± 4.4	11.8 ± 3.2	11.2 ± 4.7	12.5 ± 3.4	0.4	0.824 ^a^	–	–
eGFR/(mL·min^−1^·(1.73 m^2^)^−1^)	110.1 (108.7, 113.4)	102.6 (95.9, 109.4)	77.2 (69.4, 85.1)	44.9 (39.9, 51.9)	21.7 (19.2, 26.3)	6.2 (4.7, 8.1)	332.7	<0.001 ^c^	−0.98	<0.001
Serum creatinine/(μmol·L^−1^)	58.0 (52.3, 67.3)	61.8 (55.4, 72.1)	89.4 (81.1, 101.8)	136.4 (121.5, 146.7)	226.9 (185.1, 283.5)	690.5 (549.1, 846.8)	313.8	<0.001 ^c^	0.76	<0.001
Urea/(mmol·L^−1^)	4.5 (3.5, 5.2)	4.7 (4.0, 5.7)	5.8 (4.8, 7.0)	8.2 (6.6, 10.4)	14.1 (11.7, 15.8)	25.9 (20.6, 31.9)	257.6	<0.001 ^c^	0.81	<0.001
Hemoglobin/(g·L^−1^)	138.5 (125.0, 146.0)	130.0 (119.0, 140.5)	135.0 (121.0, 145.0)	124.0 (111.0, 135.0)	104.0 (94.0, 114.5)	96.5 (83.0, 105.2)	157.6	<0.001 ^c^	−0.41	<0.001
Total cholesterol/(mmol·L^−1^)	5.2 (4.7, 5.6)	5.5 (4.3, 7.1)	5.3 (4.0, 6.4)	4.2 (3.7, 5.5)	4.1 (3.4, 5.9)	3.8 (3.3, 4.7)	50.5	<0.001 ^c^	−0.35	<0.001
Triglycerides/(mmol·L^−1^)	1.3 (0.5, 1.8)	1.9 (1.5, 2.8)	1.8 (1.3, 2.4)	1.8 (1.2, 2.6)	1.8 (1.4, 2.4)	1.5 (1.0, 2.1)	13.5	0.009 ^c^	−0.19	0.001
Serum iron/(μmol·L^−1^)	15.5 (9.7, 21.3)	15.3 (9.5, 23.2)	14.4 (10.2, 18.5)	14.3 (10.7, 19.1)	13.4 (11.2, 20.4)	10.5 (7.1, 14.5)	24.1	<0.001 ^c^	−0.29	<0.001
Serum calcium/(mmol·L^−1^)	2.1 (2.0, 2.2)	2.1 (2.0, 2.2)	2.1 (2.0, 2.3)	2.2 (2.0, 2.3)	2.2 (2.1, 2.3)	2.1 (2.0, 2.3)	8.9	0.064 ^c^	0.03	0.542
Serum phosphorus/(mmol·L^−1^)	1.2 (1.0, 1.3)	1.2 (1.1, 1.3)	1.2 (1.0, 1.3)	1.2 (1.1, 1.3)	1.3 (1.2, 1.6)	1.9 (1.5, 2.2)	158.9	<0.001 ^c^	0.57	<0.001
Total MoCA score	28.0 (27.0, 29.0)	26.0 (23.8, 28.0)	26.0 (23.0, 27.0)	25.0 (22.0, 27.0)	22.0 (18.5, 26.0)	24.0 (22.0, 27.0)	9.6	0.048 ^c^	−0.14	0.013
Visuospatial/executive score	5.0 (5.0, 5.0)	4.0 (3.0, 5.0)	4.0 (3.0, 5.0)	4.0 (3.0, 5.0)	3.5 (2.0, 5.0)	4.0 (3.0, 5.0)	8.8	0.067 ^c^	−0.16	0.004
Naming score	3.0 (3.0, 3.0)	3.0 (3.0, 3.0)	3.0 (3.0, 3.0)	3.0 (3.0, 3.0)	3.0 (3.0, 3.0)	3.0 (3.0, 3.0)	4.6	0.325 ^c^	−0.09	0.044
Attention score	6.0 (5.0, 6.0)	6.0 (5.0, 6.0)	6.0 (5.0, 6.0)	6.0 (5.0, 6.0)	6.0 (5.0, 6.0)	6.0 (5.0, 6.0)	8.1	0.089 ^c^	−0.12	0.007
Language score	3.0 (2.0, 3.0)	3.0 (2.0, 3.0)	2.0 (2.0, 3.0)	2.0 (2.0, 3.0)	2.0 (2.0, 2.8)	2.0 (2.0, 3.0)	7.3	0.120 ^c^	−0.12	0.031
Abstraction score	2.0 (1.0, 2.0)	2.0 (1.0, 2.0)	2.0 (1.0, 2.0)	2.0 (1.0, 2.0)	1.0 (1.0, 2.0)	2.0 (1.0, 2.0)	6.5	0.166 ^c^	0.01	0.937
Delayed recall score	4.0 (3.0, 5.0)	3.0 (2.0, 4.0)	3.0 (1.0, 4.0)	3.0 (1.2, 4.0)	2.0 (1.0, 3.0)	3.0 (2.0, 4.0)	3.1	0.539 ^c^	0.01	0.924
Orientation score	6.0 (6.0, 6.0)	6.0 (6.0, 6.0)	6.0 (6.0, 6.0)	6.0 (6.0, 6.0)	6.0 (6.0, 6.0)	6.0 (6.0, 6.0)	4.7	0.317 ^c^	−0.10	0.052

CKD, chronic kidney disease; HCs, healthy controls; eGFR, estimated glomerular filtration rate; MoCA, Montreal Cognitive Assessment; –, linear-trend analysis not performed

^a^ One-way ANOVA.

^b^ Chi-square test.

^c^ Kruskal–Wallis test.

### Neuroimaging alterations across CKD stages

For cortical thickness and subcortical volume, ANCOVA identified significant overall group differences in 88 ROIs among HCs and CKD stages. Among these 88 ROIs, stage-wise post hoc comparisons showed no significant differences from HCs in CKD stages 1 to 3. Differences emerged in stage 4 (9 ROIs; Cohen’s *d*, −1.050 to −0.831) and became widespread in stage 5 (72 ROIs; Cohen’s *d*, −0.960 to −0.395). Regionally, cortical thinning in stage 5 was mainly observed in frontal, temporal, and insular lobes, accompanied by volume reductions in multiple subcortical regions. Linear trend analyses further demonstrated significant decreases across CKD stages in 127 ROIs (standardized *β*, −0.292 to −0.115), encompassing all ROIs that were significant in the ANCOVA (Fig. 3A; Table S2).

**Fig. 3.**
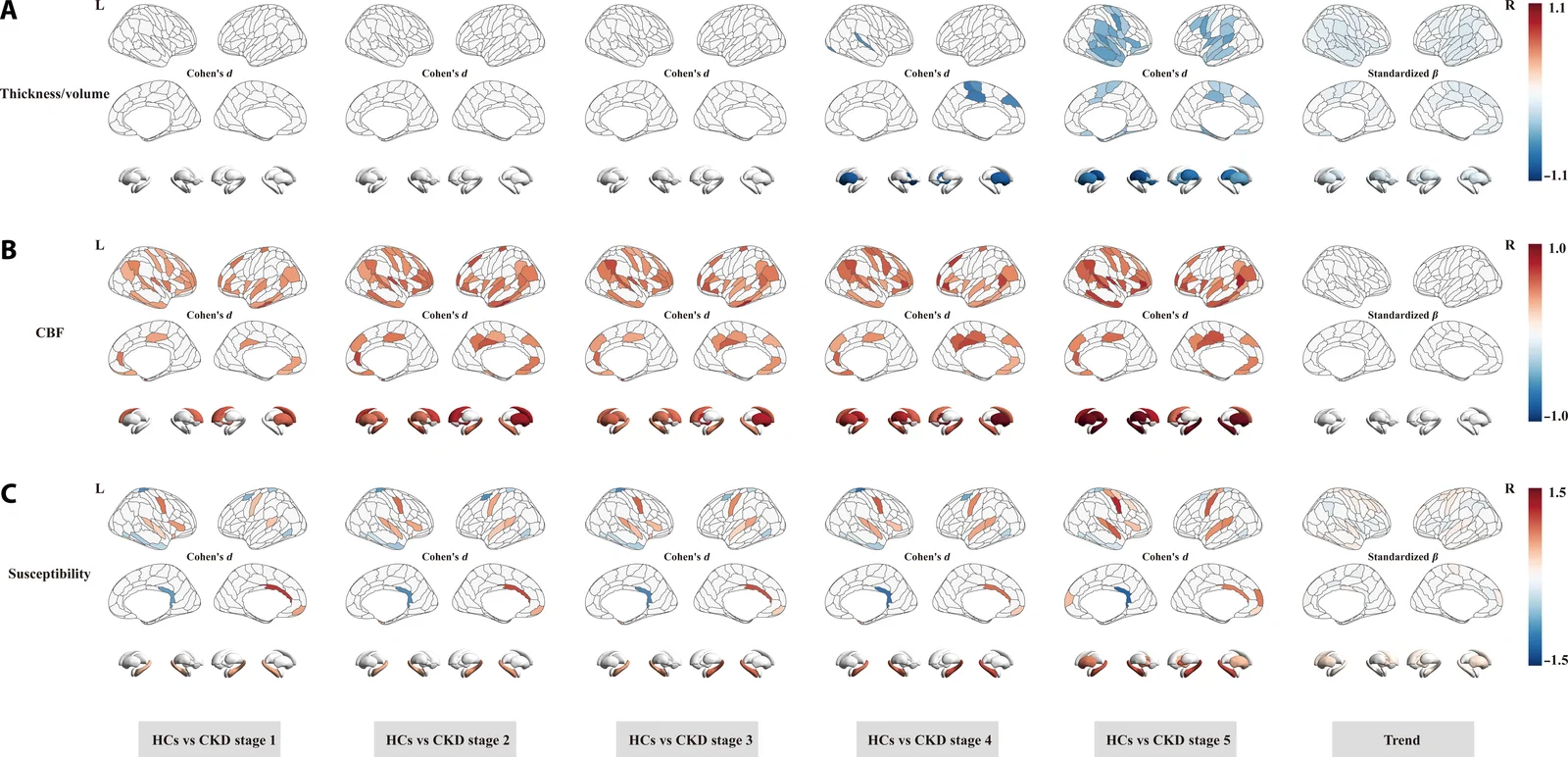
Stage-dependent multimodal neuroimaging alterations and trends across CKD stages 1 to 5. Brain surface and subcortical maps depict differences between each CKD stage and HCs and linear trends across CKD stages 1 to 5 for (A) cortical thickness/subcortical volume, (B) CBF, and (C) susceptibility. Stage-wise group differences are expressed as Cohen’s *d*, with warm and cool colors indicating higher and lower values in CKD relative to HCs, respectively. Linear trends are expressed as standardized *β* coefficients, with warm and cool colors indicating increasing and decreasing trends across CKD stages, respectively. Only regions with FDR-corrected *P* <0.05 are displayed. L, left; R, right; FDR, false discovery rate.

For CBF, ANCOVA revealed significant overall group differences in 66 ROIs. Among these 66 ROIs, stage-wise post hoc comparisons showed significant differences from HCs beginning in CKD stage 1 and persisting across all CKD stages (stage 1: 49 ROIs; stage 2: 66; stage 3: 66; stage 4: 64; stage 5: 66; Cohen’s *d*, 0.425 to 0.993), indicating a predominantly elevated CBF pattern. Regionally, CBF increases were distributed across frontal, parietal, temporal, occipital, and insular lobes as well as multiple subcortical regions. No monotonic linear trend across CKD stages was observed (Fig. 3B; Table S3).

For susceptibility, 59 ROIs showed significant overall group differences among HCs and CKD stages. Among these 59 ROIs, stage-wise comparisons identified significant susceptibility alterations across all CKD stages (stage 1: 22 ROIs; stage 2: 24; stage 3: 24; stage 4: 23; stage 5: 35; Cohen’s *d*, −1.495 to 1.457), indicating bidirectional regional changes. These susceptibility differences were predominantly located in the frontal and temporal lobes and the hippocampus. Linear trend analyses demonstrated significant increases and decreases in 73 ROIs (standardized *β*, −0.319 to 0.282), encompassing all ROIs that were significant in the ANCOVA (Fig. 3C; Table S4).

### Neuroimaging differences between CKD-NCI and CKD-CI

Compared with CKD-NCI patients, CKD-CI patients exhibited significant abnormalities in structural and susceptibility features, whereas no significant CBF differences were observed. Specifically, CKD-CI patients showed reduced cortical thickness in the bilateral superior temporal gyrus (STG_L_6_5 and STG_R_6_5) and insula (INS_L_6_1, INS_R_6_1, INS_R_6_4, INS_L_6_5, INS_R_6_5, INS_L_6_6, and INS_R_6_6). Subcortical volume was decreased in the pallidum (PAL_L and PAL_R), putamen (PUT_L and PUT_R), nucleus accumbens (NAcc_L and NAcc_R), and thalamus (THA_L and THA_R). Susceptibility was decreased in the insula (INS_L_6_5, INS_R_6_5, and INS_L_6_6), but increased in the pallidum (PAL_L and PAL_R) and putamen (PUT_L and PUT_R) (Fig. 4; Table S5). Notably, all 24 cognition-related neuroimaging features were derived from features showing significant linear trends across CKD stages.

**Fig. 4.**
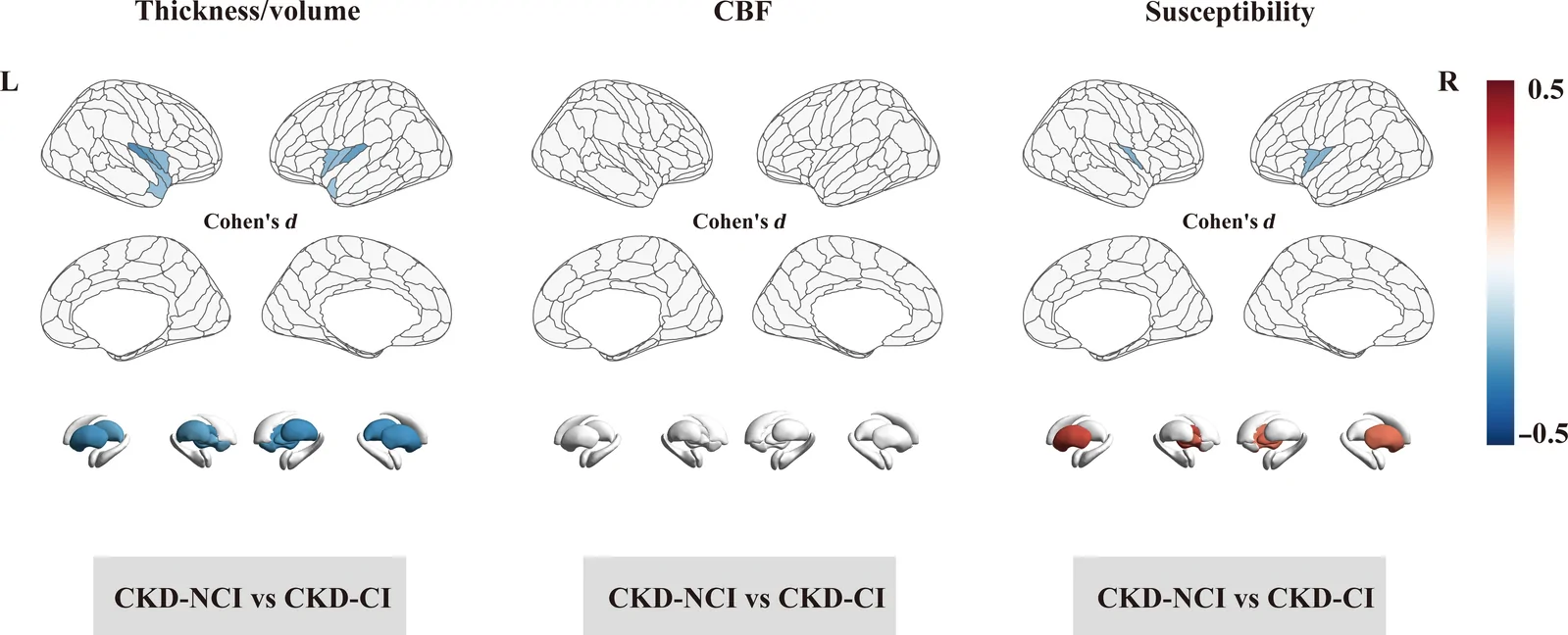
Multimodal neuroimaging differences between CKD-CI and CKD-NCI. Brain surface and subcortical maps depict comparisons between CKD-CI and CKD-NCI for cortical thickness/subcortical volume, CBF, and susceptibility. Group differences are expressed as Cohen’s *d*, with warm and cool colors indicating higher and lower values in CKD-CI relative to CKD-NCI, respectively. Only regions with FDR-corrected *P* <0.05 are displayed.

### Correlations and mediation analyses between clinical measures and cognitive performance

In the total CKD group, the total MoCA score was negatively correlated with urea (*r* = −0.157, *P* = 0.041). The visuospatial/executive score was positively associated with eGFR (*r* = 0.139, *P* = 0.028) and negatively associated with serum creatinine (*r* = −0.144, *P* = 0.028), urea (*r* = −0.150, *P* = 0.028), and serum phosphorus (*r* = −0.139, *P* = 0.028). Similarly, the attention score was positively correlated with eGFR (*r* = 0.146, *P* = 0.026) and negatively correlated with serum creatinine (*r* = −0.155, *P* = 0.023), urea (*r* = −0.162, *P* = 0.023), and serum phosphorus (*r* = −0.140, *P* = 0.027) (Fig. 5A).

**Fig. 5.**
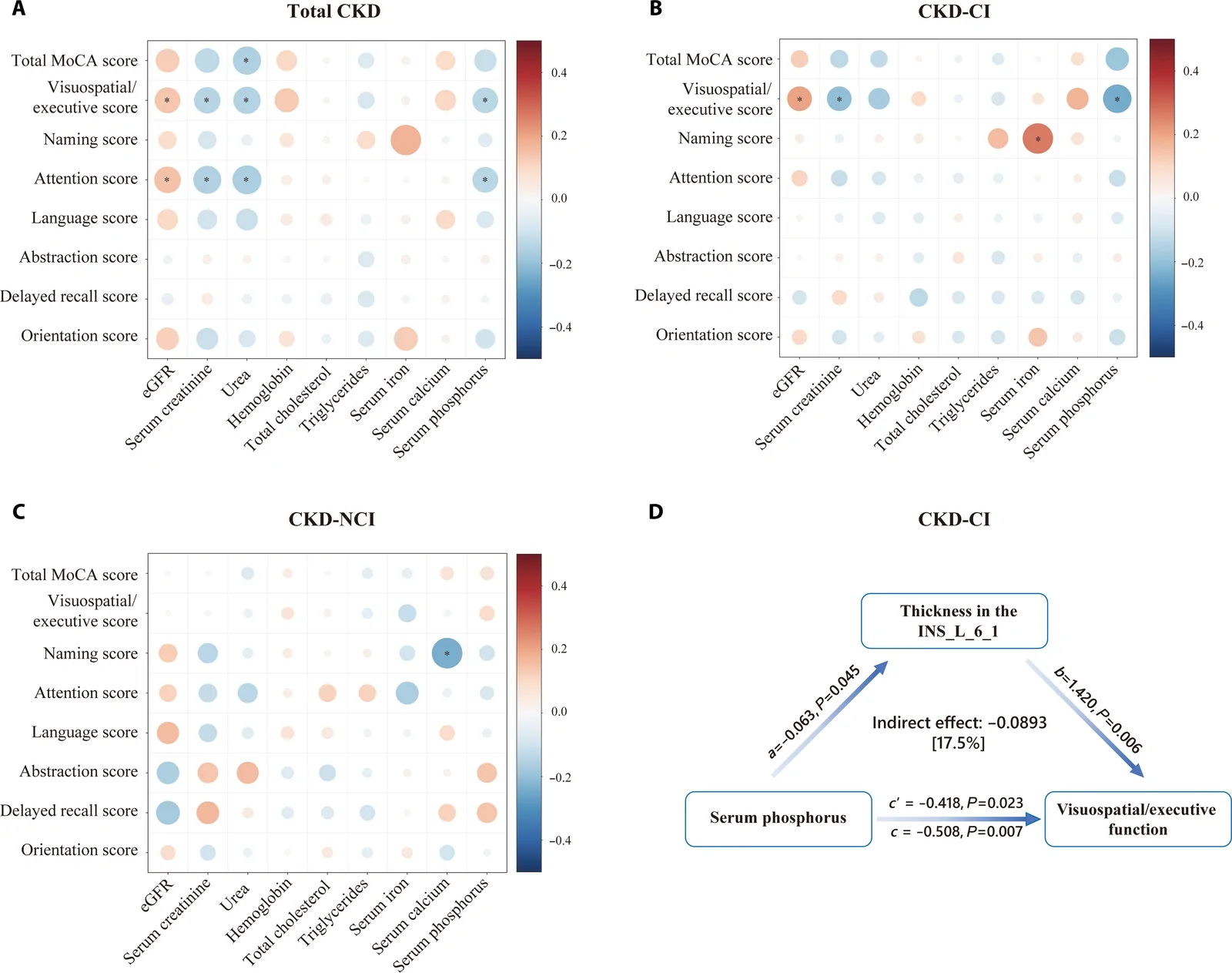
Clinical–cognitive associations and mediation analysis. (A to C) Spearman partial correlations between clinical variables and MoCA scores in the (A) total CKD group, (B) CKD-CI group, and (C) CKD-NCI group. (D) Mediation analysis of the association between serum phosphorus and visuospatial/executive performance, with cortical thickness in the INS_L_6_1 as the mediator. MoCA, Montreal Cognitive Assessment; eGFR, estimated glomerular filtration rate.

Within the CKD-CI group, the visuospatial/executive score was positively correlated with eGFR (*r* = 0.204, *P* = 0.026) and negatively correlated with serum creatinine (*r* = −0.203, *P* = 0.026) and serum phosphorus (*r* = −0.246, *P* = 0.013) (Fig. 5B). In addition, the naming score was positively associated with serum iron levels (*r* = 0.267, *P* = 0.029). In the CKD-NCI group, the naming score was negatively correlated with serum calcium levels (*r* = −0.241, *P* = 0.021) (Fig. 5C).

Within the CKD-CI group, mediation analysis showed a significant indirect association between serum phosphorus and visuospatial/executive performance through cortical thickness in the INS_L_6_1 (indirect effect = −0.089,3; 95% confidence interval, −0.209,5 to −0.004) (Fig. 5D).

### Model performance

In the discovery cohort, DBFG-Net achieved an AUC of 0.942, with an accuracy of 0.918, sensitivity of 0.904, specificity of 0.933, precision of 0.936, and an F1 score of 0.920. The Brier score was 0.068. The confusion matrix demonstrated balanced classification performance with relatively low false-positive and false-negative rates.

DBFG-Net showed higher discrimination than all baseline models, whose AUCs ranged from 0.874 to 0.912. The differences in AUC were statistically significant for all baseline models. DBFG-Net also showed the lowest Brier score among the evaluated models. DCA demonstrated a favorable net benefit for DBFG-Net across a broad range of threshold probabilities (Fig. 6A to D; Table 2).

**Fig. 6.**
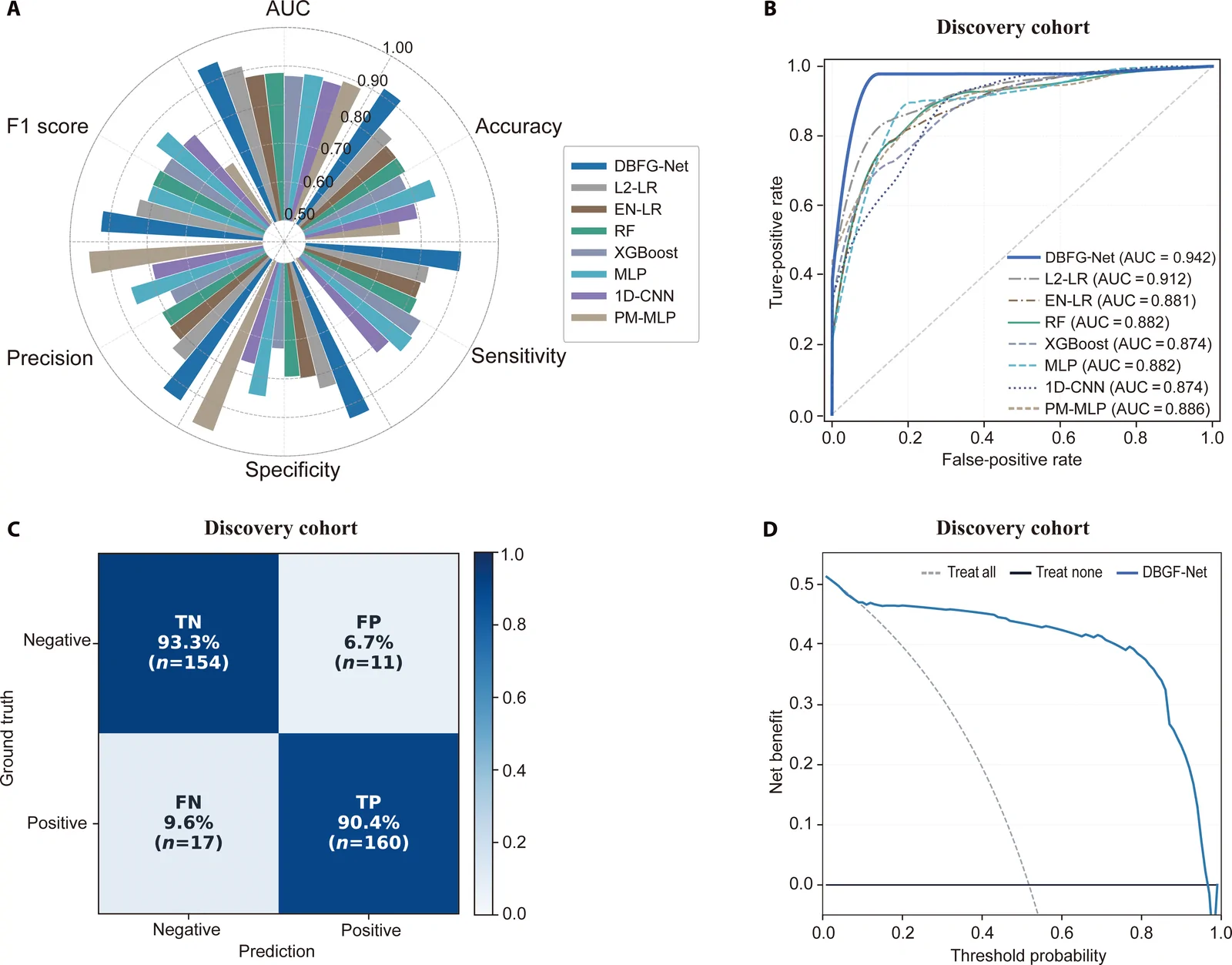
Performance of DBFG-Net and baseline models in the discovery cohort. (A) Radar plot comparing model performance. (B) ROC curves of DBFG-Net and baseline models. (C) Confusion matrix of DBFG-Net. (D) Decision curve analysis of DBFG-Net. TN, true negative; FP, false positive; FN, false negative; TP, true positive.

**Table 2.** Comparison of performance between DBFG-Net and baseline models in the discovery cohort. Values are reported as point estimates with 95% confidence intervals. The DeLong test was used to compare the AUC of DBFG-Net with that of each baseline model.

	AUC	Accuracy	Sensitivity	Specificity	Precision	F1 score	Brier score	DeLong *P*
DBFG-Net	0.942 (0.910–0.967)	0.918 (0.889–0.944)	0.904 (0.859–0.942)	0.933 (0.892–0.969)	0.936 (0.894–0.971)	0.920 (0.888–0.947)	0.068 (0.048–0.092)	–
L2-LR	0.912 (0.877–0.942)	0.830 (0.789–0.868)	0.825 (0.768–0.876)	0.836 (0.781–0.890)	0.844 (0.793–0.897)	0.834 (0.789–0.875)	0.235 (0.221–0.247)	0.027
EN-LR	0.881 (0.843–0.917)	0.807 (0.763–0.851)	0.814 (0.755–0.867)	0.800 (0.737–0.861)	0.814 (0.755–0.869)	0.814 (0.766–0.857)	0.233 (0.221–0.246)	<0.001
RF	0.882 (0.845–0.917)	0.807 (0.763–0.845)	0.819 (0.758–0.870)	0.794 (0.735–0.853)	0.810 (0.750–0.866)	0.815 (0.766–0.856)	0.169 (0.158–0.181)	<0.001
XGBoost	0.874 (0.835–0.908)	0.789 (0.746–0.830)	0.853 (0.799–0.904)	0.721 (0.649–0.784)	0.766 (0.705–0.821)	0.807 (0.760–0.846)	0.152 (0.136–0.170)	<0.001
MLP	0.882 (0.844–0.915)	0.854 (0.816–0.889)	0.859 (0.809–0.909)	0.848 (0.788–0.897)	0.859 (0.802–0.905)	0.859 (0.821–0.895)	0.149 (0.131–0.169)	0.002
1D-CNN	0.874 (0.835–0.911)	0.795 (0.751–0.839)	0.819 (0.759–0.881)	0.770 (0.706–0.830)	0.792 (0.735–0.849)	0.806 (0.758–0.851)	0.187 (0.177–0.196)	<0.001
PM-MLP	0.886 (0.850–0.919)	0.746 (0.699–0.792)	0.537 (0.462–0.611)	0.970 (0.942–0.994)	0.950 (0.904–0.989)	0.686 (0.618–0.747)	0.137 (0.123–0.149)	<0.001

AUC, area under the receiver operating characteristic curve; DBFG-Net, Dual-Branch Feature-Gated Network; L2-LR, L2-regularized logistic regression; EN-LR, elastic-net logistic regression; RF, random forest; XGBoost, extreme gradient boosting; MLP, multilayer perceptron; 1D-CNN, one-dimensional convolutional neural network; PM-MLP, parameter-count-matched multilayer perceptron; –, not applicable

### External validation of DBFG-Net

DBFG-Net maintained good discriminatory performance in both external validation cohorts. In the temporal external validation cohort, the model achieved an AUC of 0.902, accuracy of 0.913, sensitivity of 0.810, specificity of 0.984, precision of 0.971, F1 score of 0.883, and Brier score of 0.079. In the multicenter external validation cohort, the corresponding values were 0.926, 0.908, 0.848, 0.945, 0.907, 0.876, and 0.087, respectively. Confusion matrices further confirmed robust classification performance across the external validation cohorts (Fig. 7A, B, D, and E; Tables S7 and S8).

**Fig. 7.**
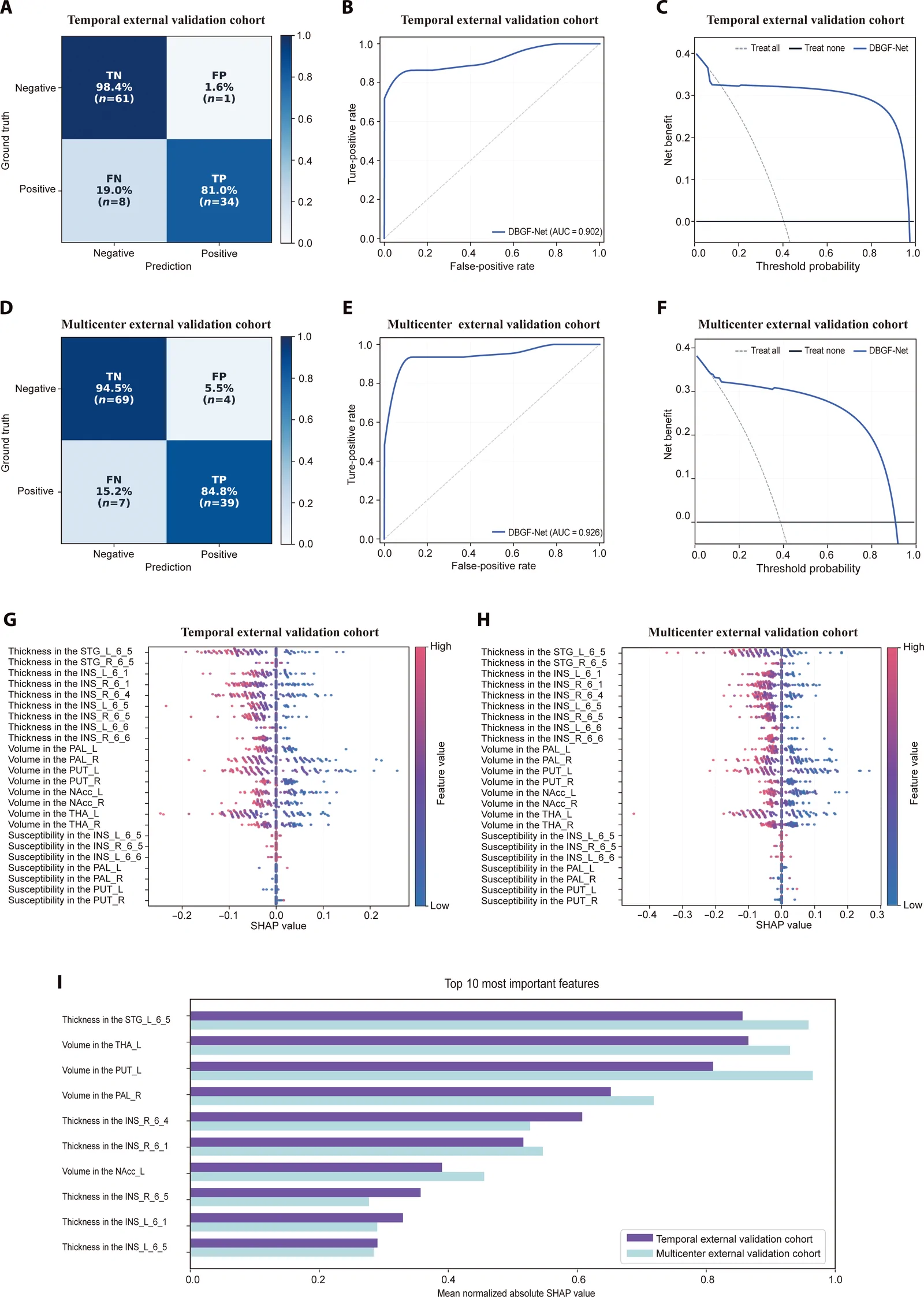
External validation and interpretability of DBFG-Net. (A to C) Confusion matrix, ROC curve, and decision curve analysis in the temporal external validation cohort. (D to F) Confusion matrix, ROC curve, and decision curve analysis in the multicenter external validation cohort. (G and H) SHAP summary plots for the temporal and multicenter external validation cohorts, respectively. (I) Top 10 features in the 2 external validation cohorts based on mean normalized absolute SHAP values. SHAP, SHapley Additive exPlanations; STG, superior temporal gyrus; INS, insula; PAL, pallidum; PUT, putamen; NAcc, nucleus accumbens; THA, thalamus.

DBFG-Net achieved the highest accuracy, specificity, precision, and F1 score among the evaluated models in both cohorts and also had the lowest Brier score. DCA demonstrated favorable net benefit across a broad range of threshold probabilities in both cohorts (Fig. 7C and F).

### Additional model analyses

Architectural ablation analyses showed that removal of the feature-recalibration module or gated fusion module reduced model discrimination, with AUCs decreasing from 0.942 to 0.850 (*P* < 0.001) and 0.907 (*P* = 0.031), respectively. In contrast, removal of the residual MLP blocks had little effect on AUC (0.938; *P* = 0.742) (Table S9).

Input-modality ablation showed that the structure-only model achieved an AUC of 0.841, whereas the susceptibility-only model showed limited discrimination (AUC, 0.522). Both single-modality models performed significantly worse than the full DBFG-Net (both *P* < 0.001) (Table S10).

After adjustment for CKD severity, DBFG-Net retained good discrimination, with an AUC of 0.892, although this was significantly lower than that of the original model (*P* < 0.001) (Table S11).

Clinical-only models showed lower discrimination than DBFG-Net, with AUCs ranging from 0.758 to 0.790. Clinical-imaging RF and XGBoost achieved AUCs of 0.941 and 0.942, respectively, which were comparable to DBFG-Net alone, whereas clinical-imaging L2-LR achieved a lower AUC of 0.912 (*P* = 0.028) (Table S12).

DBFG-Net also showed stable performance across the 10 prespecified random seeds. The mean AUC was 0.942 ± 0.007 with the fixed 5-fold allocation and 0.944 ± 0.010 when stratified 5-fold partitions were regenerated, with limited variability across the other performance metrics (Table S13).

### Model interpretability

SHAP analyses showed broadly consistent patterns of feature contribution across the 2 external validation cohorts. Lower cortical thickness and volume values generally contributed to predictions toward CKD-CI. Susceptibility features showed smaller and more heterogeneous directional contributions (Fig. 7G and H). The top 10 most important features were predominantly structural measures (Fig. 7I).

## Discussion

In this study, we developed a stage- and cognition-informed neuroimaging feature-selection framework to support the deep-learning-based identification of CKD-CI. Across the full spectrum of CKD, stage-related linear trends were observed in structural morphology and tissue susceptibility, but not in CBF. A similar modality-specific pattern was observed in the CKD-CI versus CKD-NCI comparison, in which discriminative features were confined to structural and susceptibility measures. Using features selected through this 2-step strategy, the proposed deep-learning model achieved stable performance in identifying CKD-CI.

A key finding of this study is that stage-related neuroimaging patterns differed substantially across modalities. Structural morphology and tissue susceptibility showed clear stage-dependent linear trends, whereas CBF did not show a significant monotonic stage-related trend. More advanced CKD stages were associated with lower cortical thickness and subcortical volume, which may reflect cumulative brain injury. Uremic toxin exposure, chronic neuroinflammation, and blood–brain barrier disruption may represent potential contributing mechanisms [28–30]. Prior longitudinal studies and Mendelian randomization analyses have similarly linked declining kidney function to brain atrophy [31,32]. Importantly, our stage-dependent findings extend recent QSM investigations in CKD by showing that susceptibility alterations become more pronounced with advancing disease stage. Such changes may be associated with disruptions in iron homeostasis and myelin integrity. In contrast, CBF did not show a significant monotonic trend across CKD stages. However, this finding does not exclude a role for cerebral perfusion in CKD-related brain dysfunction. Previous studies in CKD have demonstrated heterogeneous regional CBF alterations, including increased perfusion in some regions and reduced perfusion in others [11,33]. CBF may also be influenced by cerebral inflow hemodynamics [34]. These findings suggest that cerebral perfusion may reflect complex physiological and metabolic changes. Structural and susceptibility measures showed more consistent associations with CKD stage and may better reflect the cumulative burden of brain alterations in CKD [35,36]. In the present study, these features were therefore retained for CKD-CI identification.

When comparing the CKD-CI and CKD-NCI subgroups, significant differences were again confined to structural morphology and tissue susceptibility, whereas no significant CBF differences were observed. Importantly, the features distinguishing CKD-CI from CKD-NCI were entirely nested within the broader set of features exhibiting stage-dependent linear trends. This finding suggests that the neural substrates of CI may represent a cognition-relevant subset of stage-associated brain alterations across the CKD spectrum. Anatomically, the discriminative features involved several cortical and subcortical regions, including the STG, INS, PAL, PUT, NAcc, and THA, which are broadly implicated in language processing, salience detection, and cognitive control [37–39]. Among these regions, the INS may be particularly relevant, as it serves as a key hub of the salience network and supports attention engagement, resource allocation, and coordination of executive networks [40,41]. Furthermore, we evaluated associations between clinical indicators and cognitive performance in patients with CKD, while exploring potential neuroimaging mediators. In the full CKD cohort, more severe renal dysfunction and disturbances in phosphorus metabolism were associated with poorer visuospatial/executive and attentional performance, and these associations were particularly pronounced in the CKD-CI subgroup. This aligns with evidence that executive and attentional functions are especially vulnerable to uremia [5,42]. Elevated phosphate levels have been linked to endothelial dysfunction, vascular calcification, and neuroinflammation, all of which may adversely affect brain structure and executive function [43,44]. Mediation analyses further showed that cortical thickness in the left INS significantly mediated the association between serum phosphorus and visuospatial/executive performance. Given the cross-sectional design, this finding should be regarded as an exploratory statistical association rather than evidence of a causal biological pathway.

Building on the stage- and cognition-informed feature-selection framework, we developed DBFG-Net to integrate structural and susceptibility information for CKD-CI identification. Unlike previous approaches that select imaging features primarily according to cognitive status [45,46], our framework additionally incorporated stage-related changes associated with CKD severity. This strategy prioritized imaging features associated with both CKD stage and cognitive status rather than isolated group differences. DBFG-Net maintained good discrimination in the discovery cohort and 2 independent external validation cohorts. Ablation analyses supported the contribution of feature recalibration and gated fusion. Modality ablation indicated that structural measures provided the dominant discriminatory signal and susceptibility features contributed mainly as complementary information. Importantly, model performance declined after adjusting for CKD stage but remained substantial. This suggests that disease severity contributed to, but did not fully explain, the discriminative pattern. The substantially lower performance of clinical-only models suggests that the selected neuroimaging features captured discriminative information not fully represented by routine clinical measures. SHAP analysis further indicated that structural features contributed prominently to model predictions, with lower cortical thickness generally shifting predictions toward CKD-CI. Susceptibility features showed smaller and more variable SHAP contributions, possibly reflecting region-specific susceptibility alterations. From a clinical perspective, the positive net benefit observed in DCA suggests that the model may complement cognitive screening.

This study has several limitations. First, although we observed a stage-dependent pattern of brain abnormalities in CKD, the cross-sectional design cannot capture individual longitudinal trajectories or support causal inference. These findings should be validated in longitudinal follow-up studies. Second, CKD stages are clinically defined categories rather than biologically equidistant levels. Future studies using continuous eGFR and nonlinear modeling may further evaluate the robustness of the stage-related imaging findings. Third, CKD-CI was operationally defined using a MoCA cutoff rather than a comprehensive clinical and neuropsychological assessment. Although the standard education adjustment was applied, some degree of misclassification may remain, particularly among participants with scores close to the cutoff. Fourth, although all centers used the same 3.0-T scanner model, residual heterogeneity related to participant characteristics, acquisition environment, physiological status, and site-specific implementation cannot be completely excluded. Finally, the overall sample size remained modest, which may limit the precision of performance estimates. Although independent temporal and multicenter external validation supported the generalizability of the model, larger prospective multicenter cohorts are required to confirm model robustness.

## Conclusion

The stage- and cognition-informed framework identified structural and susceptibility features relevant to CKD-CI. DBFG-Net demonstrated robust performance for CKD-CI identification across the discovery and external validation cohorts.

## Data Availability

The data supporting the findings of this prospective study are not publicly available due to patient privacy and ethical restrictions. The data may be made available from the corresponding author upon reasonable request and with permission from the institutional review board.
