# Supplementary material for: Deep Learning to Identify Cognitive Impairment Across the Full Spectrum of Chronic Kidney Disease: Stage- and Cognition-Informed Feature Selection with a Dual-Branch Feature-Gated Network
Source: Cyborg Bionic Syst. 2026 Nov 6;7:0733. doi: 10.34133/cbsystems.0733 (PMC13740328; doi:10.34133/cbsystems.0733)
Supplement: Supplementary file 1 [file cbsystems.0733.f1.zip › Supplementary.docx]

**Supplementary Materials**

**Contents**

**Supplementary Methods**

Participants

MRI data acquisition

External validation and comparative models

Statistical analysis

**Supplementary Results**

Demographic, clinical, and cognitive characteristics between CKD-NCI and CKD-CI groups

**Supplementary Tables provided as separate Excel files**

Supplementary Table S1. Brainnetome atlas-based ROI definitions used in this study (210 cortical regions and 14 merged subcortical regions)

Supplementary Table S2. ROI-wise ANCOVA results for structural measures and post hoc stage-wise comparisons versus HCs, with linear trend analyses across CKD stages

Supplementary Table S3. ROI-wise ANCOVA results for CBF and post hoc stage-wise comparisons versus HCs, with linear trend analyses across CKD stages

Supplementary Table S4. ROI-wise ANCOVA results for susceptibility and post hoc stage-wise comparisons versus HCs, with linear trend analyses across CKD stages

Supplementary Table S5. Neuroimaging differences between CKD-NCI and CKD-CI

**Supplementary Tables included in this document**

Supplementary Table S6. Demographic, clinical, and cognitive characteristics between CKD-NCI and CKD-CI groups

Supplementary Table S7. Performance comparison of DBFG-Net and baseline models in the temporal external validation cohort

Supplementary Table S8. Performance comparison of DBFG-Net and baseline models in the multicenter external validation cohort

Supplementary Table S9. Architectural ablation analysis of DBFG-Net

Supplementary Table S10. Input-modality ablation analysis of DBFG-Net

Supplementary Table S11. Sensitivity analysis of DBFG-Net adjusted for disease severity

Supplementary Table S12. Comparison of imaging-only, clinical-only, and clinical-imaging models

Supplementary Table S13. Stability analysis of DBFG-Net across 10 prespecified random seeds

**Supplementary Methods**

**Participants**

All cohorts adhered to consistent inclusion/exclusion criteria, clinical data collection, and magnetic resonance imaging (MRI) sequences (as detailed in the main text). Healthy controls (HCs) were screened without kidney, neurological, psychiatric, or major systemic diseases and matched for age and sex, using the same exclusion criteria as chronic kidney disease (CKD) patients. Below are the specific enrollment timelines, participating centers, recruitment methods, total assessed participants, exclusion reasons with counts, and final included numbers for each cohort.

Discovery cohort (CKD Patients): Enrollment occurred from March 2023 to October 2024 at the Department of Nephrology, Beijing Friendship Hospital, Capital Medical University. A total of 385 CKD patients were assessed. Exclusions included stroke (n = 6), MRI contraindications (n = 11), poor MRI quality (n = 9), and incomplete imaging data (n = 17), for a total of 43 exclusions. This resulted in 342 included patients, subdivided into CKD with cognitive impairment (CKD-CI) (n = 177) and CKD without cognitive impairment (CKD-NCI) (n = 165) based on Montreal Cognitive Assessment scores.

Discovery cohort (HCs): Enrollment was concurrent with CKD patients, recruited via community advertisement. A total of 60 HCs were assessed and screened using the same exclusion criteria as CKD participants; HCs underwent the same MRI and neuropsychological protocols. Exclusions included poor MRI quality (n = 2), for a total of 2 exclusions. This resulted in 58 included HCs.

Temporal validation cohort: Enrollment occurred from November 2024 to November 2025 at Beijing Friendship Hospital, Capital Medical University (same center as discovery cohort). Patients were prospectively enrolled via hospital referrals. A total of 111 CKD patients were assessed. Exclusions included MRI contraindications (n = 2), poor MRI quality (n = 3), and incomplete imaging data (n = 2), for a total of 7 exclusions. This resulted in 104 included patients, subdivided into CKD-CI (n = 42) and CKD-NCI (n = 62).

Multicenter validation cohort: Enrollment occurred from June 2024 to August 2025 at two independent centers: Binzhou Medical University Hospital and Linyi Hospital of Traditional Chinese Medicine. A total of 127 CKD patients were assessed. Exclusions included poor MRI quality (n = 5) and incomplete imaging data (n = 3), for a total of 8 exclusions. This resulted in 119 included patients, subdivided into CKD-CI (n = 46) and CKD-NCI (n = 73).

**MRI data acquisition**

T1-Weighted structural imaging: Acquired using a high-resolution 3D brain volume sequence with repetition time (TR)/echo time (TE) = 8.8/3.5 ms, inversion time (TI) = 450 ms, flip angle (FA) = 15°, acquisition matrix = 256 × 256, field of view (FOV) = 240 × 240 mm², voxel size = 1.0 × 1.0 × 1.0 mm³, and number of slices = 196 with no interslice gap.

Perfusion imaging: Acquired using a 3D pseudo-continuous arterial spin labeling sequence with TR/TE = 5316/10.7 ms, labeling time = 2025 ms, post-label delay = 2025 ms, FA = 111°, FOV = 240 × 240 mm², number of in-plane spiral arm = 8, points per arm = 512, slice thickness = 4 mm, and number of slices = 36 with no interslice gap. Additionally, a proton density-weighted image (M0) was acquired to enable quantitative CBF calculation.

Quantitative susceptibility mapping: Acquired using a 3D multi-echo gradient echo sequence with TR = 23.1 ms, first TE = 3.4 ms, ∆TE = 2.3 ms, number of echoes = 8, FA = 12°, acquisition matrix = 256 × 256, FOV = 256 × 256 mm², slice thickness = 1.0 mm, and number of axial slices = 140 with no interslice gap.

**External validation and** **comparative models**

Machine learning models used scikit-learn (v1.6.1) and the XGBoost library (v3.0.5), whereas deep learning models were built in PyTorch. All comparative models were implemented using the same data partitioning and preprocessing framework as DBFG-Net. During internal validation, all models were trained and evaluated using the same fold-specific selected features and held-out test participants as DBFG-Net. For external validation, all comparative models were retrained using the final 24-feature set derived from the complete discovery cohort and were subsequently evaluated in the independent external validation cohorts.

The conventional machine-learning models included L2-regularized logistic regression (L2-LR), elastic-net logistic regression (EN-LR), random forest (RF), and extreme gradient boosting (XGBoost).

L2-LR was implemented using the logistic regression module in scikit-learn v1.6.1 with an L2 penalty, 𝐶=0.0001, the liblinear solver, class-balanced weighting, and a maximum of 3,000 iterations.

EN-LR was implemented using the saga solver with class-balanced weighting and a maximum of 10,000 iterations. The final EN-LR configuration used 𝐶 =0.01 and an L1 ratio of 0.7.

RF was implemented with 20 estimators, a maximum tree depth of 3, a minimum of 20 samples per leaf, square-root feature sampling, and class-balanced weighting.

XGBoost was implemented with 50 estimators, a learning rate of 0.05, a maximum tree depth of 2, subsample and column-sampling ratios of 0.6, 𝐿1 regularization coefficient (𝑟𝑒𝑔_𝑎𝑙𝑝ℎ𝑎) of 1.0, and 𝐿2 regularization coefficient (𝑟𝑒𝑔_𝑙𝑎𝑚𝑏𝑑𝑎) of 1.0.

The neural-network baselines included a multilayer perceptron (MLP), a one-dimensional convolutional neural network (1D-CNN), and a parameter-count-matched MLP (PM-MLP).

The MLP baseline consisted of a trainable global feature-weighting layer followed by a fully connected network with hidden dimensions of 8 and 4. During internal validation, the input dimension was adapted according to the fold-specific selected feature number, resulting in a 𝐷_𝑘_-8-4-1 architecture. Batch normalization, ReLU activation, and dropout of 0.25 were applied. The model was trained using Adam with a batch size of 8, a learning rate of 0.3, weight decay of 0.02, a maximum of 50 epochs, and early stopping with a patience of 20 epochs. Gaussian noise with a standard deviation of 0.3 was added to the input features during training.

The 1D-CNN baseline used a trainable global feature-weighting layer followed by grouped 1×1 convolution with a hidden dimension of 8. The model used dropout of 0.5 and Gaussian input noise with a standard deviation of 0.5. Training was performed using Adam with a batch size of 8, a learning rate of 0.1, weight decay of 0.01, a maximum of 50 epochs, and early stopping with a patience of 2 epochs.

The PM-MLP baseline was designed as a capacity-matched control for DBFG-Net. In the final 24-feature configuration, the model consisted of a trainable global feature-weighting layer followed by a fully connected network with dimensions of 24-172-86-1. Both hidden layers used batch normalization, ReLU activation, and dropout of 0.25. The final PM-MLP contained 19805 trainable parameters, which was comparable to the 19646 parameters of DBFG-Net. During internal validation, the input layer was adjusted according to the fold-specific feature number while maintaining the same single-stream architecture. Training was performed using Adam with a batch size of 8, a learning rate of 0.3, weight decay of 0.02, and Gaussian input noise with a standard deviation of 0.3.

**Statistical analysis**

Group differences between CKD-CI and CKD-NCI were assessed using appropriate statistical tests. Normally distributed continuous variables were compared using the independent-samples *t* test. Non-normally distributed continuous variables were compared using the Mann–Whitney *U* test, and categorical variables were compared using the chi-square test. *P*<0.05 was considered statistically significant.

**Supplementary Results**

**Demographic, clinical, and cognitive characteristics between CKD-NCI and CKD-CI groups**

Compared with the CKD-NCI group, the CKD-CI group was older and had fewer years of education. The CKD-CI group also showed lower eGFR, hemoglobin, and serum calcium levels, and higher serum creatinine and urea levels. No significant differences were observed in sex, total cholesterol, triglycerides, serum iron, or serum phosphorus (**Supplementary Table S6**).

**Supplementary Table S1.** Brainnetome atlas–based ROI definitions used in this study (210 cortical regions and 14 merged subcortical regions)

Provided as a separate Excel file due to the large volume of data.

**Supplementary Table S2.** ROI-wise ANCOVA results for structural measures and post hoc stage-wise comparisons versus HCs, with linear trend analyses across CKD stages

Provided as a separate Excel file due to the large volume of data.

**Supplementary Table S3.** ROI-wise ANCOVA results for CBF and post hoc stage-wise comparisons versus HCs, with linear trend analyses across CKD stages

Provided as a separate Excel file due to the large volume of data.

**Supplementary Table S4.** ROI-wise ANCOVA results for susceptibility and post hoc stage-wise comparisons versus HCs, with linear trend analyses across CKD stages

Provided as a separate Excel file due to the large volume of data.

**Supplementary Table S5.** Neuroimaging differences between CKD-NCI and CKD-CI

Provided as a separate Excel file due to the large volume of data.

**Supplementary Table S6.** Demographic, clinical, and cognitive characteristics between CKD-NCI and CKD-CI groups

| Characteristic | CKD-NCI  (n=165) | CKD-CI  (n=177) | Test  statistic | P |
| --- | --- | --- | --- | --- |
| Age (year) | 45.4 ± 11.6 | 58.4 ± 10.9 | -10.9 | **<0.001**^a^ |
| Gender (Male/Female) | 107/58 | 105/72 | 1.1 | 0.293^b^ |
| Education (year) | 13.8 ± 2.8 | 10.7 ± 3.8 | 7.3 | **<0.001**^a^ |
| eGFR (mL/min/1.73 m²) | 60.8 (26.4, 88.2) | 41.3 (7.2, 79.2) | 3.3 | **<0.001**^c^ |
| Serum creatinine (μmol/L) | 111.2 (83.2, 265.2) | 142.5 (85.8, 561.7) | -2.0 | **0.045**^c^ |
| Urea (mmol/L) | 6.9 (5.0, 13.5) | 9.5 (6.2, 22.5) | -3.5 | **<0.001**^c^ |
| Hemoglobin (g/L) | 125.0 (104.0, 142.0) | 116.0 (95.0, 129.0) | 3.8 | **<0.001**^c^ |
| Total cholesterol (mmol/L) | 4.5 (3.7, 5.9) | 4.4 (3.5, 5.8) | 1.0 | 0.321^c^ |
| Triglycerides (mmol/L) | 1.8 (1.3, 2.5) | 1.7 (1.2, 2.3) | 1.7 | 0.095^c^ |
| Serum iron (μmol/L) | 13.7 (9.4, 17.7) | 11.7 (8.2, 15.4) | 1.9 | 0.054^c^ |
| Serum calcium (mmol/L) | 2.2 (2.0, 2.3) | 2.1 (2.0, 2.2) | 3.0 | **0.003**^c^ |
| Serum phosphorus (mmol/L) | 1.3 (1.1, 1.5) | 1.3 (1.1, 1.7) | -1.0 | 0.308^c^ |
| Total MoCA score | 27.0 (26.0, 28.0) | 22.5 (20.0, 24.0) | 13.9 | **<0.001**^c^ |
| Visuospatial/executive score | 5.0 (4.0, 5.0) | 3.0 (2.0, 4.0) | 9.5 | **<0.001**^c^ |
| Naming score | 3.0 (3.0, 3.0) | 3.0 (3.0, 3.0) | 5.3 | **<0.001**^c^ |
| Attention score | 6.0 (6.0, 6.0) | 5.0 (5.0, 6.0) | 7.5 | **<0.001**^c^ |
| Language score | 3.0 (2.0, 3.0) | 2.0 (2.0, 3.0) | 7.3 | **<0.001**^c^ |
| Abstraction score | 2.0 (2.0, 2.0) | 1.0 (1.0, 2.0) | 7.7 | **<0.001**^c^ |
| Delayed recall score | 4.0 (3.0, 4.0) | 2.0 (1.0, 3.0) | 11.1 | **<0.001**^c^ |
| Orientation score | 6.0 (6.0, 6.0) | 6.0 (6.0, 6.0) | 3.8 | **<0.001**^c^ |

^a^ Independent-samples t test. ^b^ Chi-square test. ^c^ Mann–Whitney U test.

Test statistic denotes the t (independent-samples t test), χ² (chi-square), or Z (Mann–Whitney U test) statistic.

Abbreviations: CKD-NCI, chronic kidney disease without cognitive impairment; CKD-CI, chronic kidney disease with cognitive impairment; eGFR, estimated glomerular filtration rate; MoCA, Montreal Cognitive Assessment.

**Supplementary Table S7.** Comparison of performance between DBFG-Net and baseline models in the temporal external validation cohort

|  | AUC | Accuracy | Sensitivity | Specificity | Precision | F1 score | Brier score | DeLong P |
| --- | --- | --- | --- | --- | --- | --- | --- | --- |
| DBFG-Net | 0.902 (0.822–0.965) | 0.913 (0.856–0.962) | 0.810 (0.684–0.919) | 0.984 (0.948–0.998) | 0.971 (0.906–0.998) | 0.883 (0.794–0.951) | 0.079 (0.037–0.129) | – |
| L2-LR | 0.812 (0.706–0.903) | 0.798 (0.712–0.875) | 0.762 (0.628–0.889) | 0.823 (0.725–0.918) | 0.744 (0.600–0.878) | 0.753 (0.630–0.848) | 0.237 (0.226–0.248) | 0.031 |
| EN-LR | 0.807 (0.705–0.895) | 0.740 (0.654–0.827) | 0.643 (0.486–0.787) | 0.806 (0.698–0.900) | 0.692 (0.547–0.830) | 0.667 (0.533–0.775) | 0.239 (0.229–0.245) | 0.013 |
| RF | 0.752 (0.656–0.839) | 0.740 (0.654–0.817) | 0.571 (0.436–0.711) | 0.855 (0.759–0.935) | 0.727 (0.562–0.871) | 0.640 (0.514–0.747) | 0.197 (0.174–0.222) | 0.001 |
| XGBoost | 0.766 (0.663–0.863) | 0.731 (0.644–0.817) | 0.690 (0.548–0.826) | 0.758 (0.649–0.862) | 0.659 (0.523–0.800) | 0.674 (0.554–0.784) | 0.192 (0.157–0.230) | 0.002 |
| MLP | 0.880 (0.786–0.952) | 0.856 (0.788–0.913) | 0.810 (0.676–0.921) | 0.887 (0.803–0.954) | 0.829 (0.703–0.929) | 0.819 (0.712–0.900) | 0.136 (0.109–0.167) | 0.441 |
| 1D-CNN | 0.833 (0.747–0.912) | 0.760 (0.683–0.837) | 0.738 (0.605–0.865) | 0.774 (0.677–0.873) | 0.689 (0.556–0.814) | 0.713 (0.597–0.812) | 0.199 (0.186–0.212) | 0.022 |
| PM-MLP | 0.823 (0.782–0.862) | 0.750 (0.692–0.817) | 0.429 (0.351–0.527) | 0.968 (0.908–0.981) | 0.900 (0.817–0.962) | 0.581 (0.507–0.670) | 0.138 (0.127–0.149) | 0.027 |

Values are reported as point estimates with 95% confidence intervals.

The DeLong test was used to compare the AUC of DBFG-Net with that of each baseline model.

Abbreviations: AUC, area under the receiver operating characteristic curve; DBFG-Net, Dual-Branch Feature-Gated Network; L2-LR, L2-regularized logistic regression; EN-LR, elastic-net logistic regression; RF, random forest; XGBoost, extreme gradient boosting; MLP, multilayer perceptron; 1D-CNN, one-dimensional convolutional neural network; PM-MLP, parameter-count-matched multilayer perceptron.

**Supplementary Table S8.** Comparison of performance between DBFG-Net and baseline models in the multicenter external validation cohort

|  | AUC | Accuracy | Sensitivity | Specificity | Precision | F1 score | Brier score | DeLong P |
| --- | --- | --- | --- | --- | --- | --- | --- | --- |
| DBFG-Net | 0.926 (0.867–0.977) | 0.908 (0.857–0.958) | 0.848 (0.738–0.943) | 0.945 (0.887–0.988) | 0.907 (0.816–0.979) | 0.876 (0.795–0.943) | 0.087 (0.041–0.133) | – |
| L2-LR | 0.831 (0.741–0.908) | 0.807 (0.739–0.874) | 0.783 (0.650–0.902) | 0.822 (0.736–0.901) | 0.735 (0.604–0.854) | 0.758 (0.650–0.848) | 0.236 (0.225–0.247) | 0.002 |
| EN-LR | 0.832 (0.743–0.907) | 0.748 (0.664–0.815) | 0.783 (0.654–0.902) | 0.726 (0.618–0.829) | 0.643 (0.509–0.760) | 0.706 (0.596–0.796) | 0.238 (0.231–0.245) | 0.002 |
| RF | 0.842 (0.758–0.911) | 0.756 (0.681–0.832) | 0.652 (0.513–0.800) | 0.822 (0.735–0.903) | 0.698 (0.561–0.825) | 0.674 (0.560–0.784) | 0.175 (0.156–0.197) | 0.006 |
| XGBoost | 0.840 (0.761–0.911) | 0.731 (0.655–0.807) | 0.739 (0.600–0.870) | 0.726 (0.623–0.831) | 0.630 (0.500–0.759) | 0.680 (0.558–0.775) | 0.169 (0.141–0.198) | 0.006 |
| MLP | 0.912 (0.841–0.968) | 0.857 (0.790–0.916) | 0.870 (0.755–0.958) | 0.849 (0.762–0.926) | 0.784 (0.660–0.891) | 0.825 (0.731–0.899) | 0.135 (0.112–0.162) | 0.341 |
| 1D-CNN | 0.903 (0.839–0.956) | 0.790 (0.714–0.857) | 0.826 (0.707–0.932) | 0.767 (0.671–0.863) | 0.691 (0.567–0.811) | 0.752 (0.644–0.839) | 0.194 (0.182–0.207) | 0.306 |
| PM-MLP | 0.827 (0.792–0.861) | 0.748 (0.669–0.820) | 0.457 (0.352–0.533) | 0.932 (0.875–0.987) | 0.808 (0.715–0.883) | 0.583 (0.501–0.676) | 0.137 (0.128–0.149) | 0.001 |

Values are reported as point estimates with 95% confidence intervals.

The DeLong test was used to compare the AUC of DBFG-Net with that of each baseline model.

Abbreviations: AUC, area under the receiver operating characteristic curve; DBFG-Net, Dual-Branch Feature-Gated Network; L2-LR, L2-regularized logistic regression; EN-LR, elastic-net logistic regression; RF, random forest; XGBoost, extreme gradient boosting; MLP, multilayer perceptron; 1D-CNN, one-dimensional convolutional neural network; PM-MLP, parameter-count-matched multilayer perceptron.

**Supplementary Table S9.** Architectural ablation analysis of DBFG-Net

|  | AUC | Accuracy | Sensitivity | Specificity | Precision | F1 score | Brier score | DeLong P |
| --- | --- | --- | --- | --- | --- | --- | --- | --- |
| DBFG-Net | 0.942 (0.910–0.967) | 0.918 (0.889–0.944) | 0.904 (0.859–0.942) | 0.933 (0.892–0.969) | 0.936 (0.894–0.971) | 0.920 (0.888–0.947) | 0.068 (0.048–0.092) | – |
| Without feature recalibration | 0.850 (0.741–0.908) | 0.778 (0.734–0.822) | 0.893 (0.841–0.936) | 0.655 (0.581–0.725) | 0.735 (0.678–0.792) | 0.806 (0.761–0.846) | 0.228 (0.224–0.232) | <0.001 |
| Without gated fusion | 0.907 (0.874–0.936) | 0.804 (0.760–0.845) | 0.870 (0.820–0.916) | 0.733 (0.665–0.800) | 0.778 (0.721–0.835) | 0.821 (0.776–0.861) | 0.224 (0.221–0.227) | 0.031 |
| Without residual blocks | 0.938 (0.908–0.962) | 0.904 (0.868–0.936) | 0.876 (0.825–0.925) | 0.933 (0.894–0.969) | 0.934 (0.894–0.970) | 0.904 (0.868–0.935) | 0.183 (0.177–0.190) | 0.742 |

Values are reported as point estimates with 95% confidence intervals.

The DeLong test was used to compare the AUC of DBFG-Net with that of each ablated variant.

Abbreviations: DBFG-Net, Dual-Branch Feature-Gated Network; AUC, area under the receiver operating characteristic curve.

**Supplementary Table S10.** Input-modality ablation analysis of DBFG-Net

|  | AUC | Accuracy | Sensitivity | Specificity | Precision | F1 score | Brier score | DeLong P |
| --- | --- | --- | --- | --- | --- | --- | --- | --- |
| DBFG-Net | 0.942 (0.910–0.967) | 0.918 (0.889–0.944) | 0.904 (0.859–0.942) | 0.933 (0.892–0.969) | 0.936 (0.894–0.971) | 0.920 (0.888–0.947) | 0.068 (0.048–0.092) | – |
| Structure-only | 0.841 (0.798–0.880) | 0.792 (0.746–0.836) | 0.921 (0.879–0.959) | 0.655 (0.578–0.726) | 0.741 (0.679–0.796) | 0.821 (0.776–0.859) | 0.206 (0.198–0.213) | <0.001 |
| Susceptibility-only | 0.522 (0.461–0.584) | 0.500 (0.447–0.553) | 0.367 (0.302–0.438) | 0.642 (0.570–0.717) | 0.524 (0.439–0.615) | 0.432 (0.365–0.500) | 0.250 (0.247–0.252) | <0.001 |

Values are reported as point estimates with 95% confidence intervals.

The DeLong test was used to compare the AUC of DBFG-Net with that of each single-modality variant.

Abbreviations: DBFG-Net, Dual-Branch Feature-Gated Network; AUC, area under the receiver operating characteristic curve.

**Supplementary Table S11.** Sensitivity analysis of DBFG-Net adjusted for disease severity

|  | AUC | Accuracy | Sensitivity | Specificity | Precision | F1 score | Brier score | DeLong P |
| --- | --- | --- | --- | --- | --- | --- | --- | --- |
| DBFG-Net | 0.942 (0.910–0.967) | 0.918 (0.889–0.944) | 0.904 (0.859–0.942) | 0.933 (0.892–0.969) | 0.936 (0.894–0.971) | 0.920 (0.888–0.947) | 0.068 (0.048–0.092) | – |
| Severity-adjusted DBFG-Net | 0.892 (0.851–0.928) | 0.860 (0.822–0.895) | 0.831 (0.776–0.885) | 0.891 (0.841–0.938) | 0.891 (0.842–0.938) | 0.860 (0.819–0.897) | 0.115 (0.089–0.142) | <0.001 |

Values are reported as point estimates with 95% confidence intervals.

The DeLong test was used to compare the AUC of DBFG-Net with that of the severity-adjusted DBFG-Net.

Abbreviations: DBFG-Net, Dual-Branch Feature-Gated Network; AUC, area under the receiver operating characteristic curve.

**Supplementary Table S12.** Comparison of imaging-only, clinical-only, and clinical–imaging models

|  | AUC | Accuracy | Sensitivity | Specificity | Precision | F1 score | Brier score | DeLong P |
| --- | --- | --- | --- | --- | --- | --- | --- | --- |
| DBFG-Net | 0.942 (0.910–0.967) | 0.918 (0.889–0.944) | 0.904 (0.859–0.942) | 0.933 (0.892–0.969) | 0.936 (0.894–0.971) | 0.920 (0.888–0.947) | 0.068 (0.048–0.092) | – |
| Clinical-only L2-LR | 0.761 (0.710–0.806) | 0.693 (0.643–0.740) | 0.661 (0.586–0.732) | 0.727 (0.663–0.789) | 0.722 (0.656–0.786) | 0.690 (0.630–0.741) | *0.238* (0.228–0.249) | <0.001 |
| Clinical-only RF | 0.758 (0.709–0.809) | 0.702 (0.652–0.749) | 0.701 (0.631–0.766) | 0.703 (0.634–0.768) | 0.717 (0.652–0.780) | 0.709 (0.653–0.759) | 0.204 (0.191–0.217) | <0.001 |
| Clinical-only XGBoost | 0.790 (0.740–0.836) | 0.737 (0.690–0.784) | 0.819 (0.759–0.877) | 0.648 (0.574–0.717) | 0.714 (0.647–0.772) | 0.763 (0.714–0.809) | 0.192 (0.176–0.209) | <0.001 |
| Clinical–imaging L2-LR | 0.912 (0.876–0.943) | 0.851 (0.807–0.886) | 0.859 (0.801–0.908) | 0.842 (0.786–0.893) | 0.854 (0.797–0.900) | 0.856 (0.813–0.892) | 0.236 (0.225–0.246) | 0.028 |
| Clinical–imaging RF | 0.941 (0.909–0.968) | 0.915 (0.883–0.942) | 0.893 (0.843–0.936) | 0.939 (0.899–0.972) | 0.940 (0.903–0.973) | 0.916 (0.883–0.943) | 0.106 (0.093–0.122) | 0.934 |
| Clinical–imaging XGBoost | 0.942 (0.913–0.967) | 0.921 (0.892–0.947) | 0.904 (0.859–0.941) | 0.939 (0.901–0.975) | 0.941 (0.909–0.976) | 0.922 (0.892–0.948) | 0.085 (0.071–0.101) | 0.984 |

Values are reported as point estimates with 95% confidence intervals.

The DeLong test was used to compare AUCs between DBFG-Net and each comparator model.

Abbreviations: AUC, area under the receiver operating characteristic curve; DBFG-Net, Dual-Branch Feature-Gated Network; L2-LR, L2-regularized logistic regression; RF, random forest; XGBoost, extreme gradient boosting.

**Supplementary Table S13.** Stability analysis of DBFG-Net across 10 prespecified random seeds

| Performance metric | Fixed folds,  Mean ± SD | Range | Regenerated stratified 5-fold partitions,  Mean ± SD | Range |
| --- | --- | --- | --- | --- |
| AUC | 0.942 ± 0.007 | 0.930–0.952 | 0.944 ± 0.010 | 0.926–0.960 |
| Accuracy | 0.922 ± 0.008 | 0.909–0.939 | 0.923 ± 0.006 | 0.912–0.930 |
| Sensitivity | 0.906 ± 0.007 | 0.893–0.915 | 0.903 ± 0.013 | 0.881–0.927 |
| Specificity | 0.939 ± 0.013 | 0.921–0.964 | 0.944 ± 0.009 | 0.933–0.964 |
| Precision | 0.941 ± 0.012 | 0.924–0.964 | 0.945 ± 0.008 | 0.936–0.963 |
| F1 score | 0.923 ± 0.008 | 0.911–0.939 | 0.924 ± 0.006 | 0.912–0.932 |
| Brier score | 0.070 ± 0.007 | 0.058–0.080 | 0.069 ± 0.005 | 0.062–0.079 |

Abbreviations: AUC, area under the receiver operating characteristic curve; DBFG-Net, Dual-Branch Feature-Gated Network; SD, standard deviation.
